# Is HbA1c a valid surrogate for mortality in type 2 diabetes? Evidence from a meta-analysis of randomized trials

**DOI:** 10.1007/s00592-022-01887-y

**Published:** 2022-05-09

**Authors:** Christina Baechle, Wiebke Scherler, Alexander Lang, Tim Filla, Oliver Kuss

**Affiliations:** 1 Institute for Biometrics and Epidemiology, German Diabetes Center, Leibniz Center for Diabetes Research at Heinrich-Heine-University Düsseldorf, Auf’m Hennekamp 65, 40225 Düsseldorf, Germany; 2 Institute of Medical Biometrics and Bioinformatics, Medical Faculty, Heinrich-Heine-University Düsseldorf, Moorenstr. 5, 40225 Düsseldorf, Germany; 3 Center for Health and Society, Medical Faculty, Heinrich-Heine-University Düsseldorf, Moorenstr. 5, 40225 Düsseldorf, Germany; 4 German Center for Diabetes Research (DZD), Ingolstädter Landstr. 1, 85764 Neuherberg, Germany

**Keywords:** HbA1c, Hemoglobin A1c, Prediction, Surrogate, Mortality, Type 2 diabetes

## Abstract

**Aims:**

Hemoglobin A1c (HbA1c) has been repeatedly questioned as a valid surrogate marker, especially for patient-relevant outcomes. The aim of this study was to validate the HbA1c value as a surrogate for all-cause mortality in people with type 2 diabetes.

**Methods:**

The effect estimates for HbA1c lowering after treatment as well as reductions in all-cause mortality of randomized trials were extracted from a systematic review and updated. For the measurement of actual surrogacy, weighted linear regression models with a random intercept for the study effect were used with the all-cause mortality estimate (risk difference and log relative risk) as the outcome and the estimate for HbA1c difference as the covariate. Surrogacy was assessed according to the criteria of Daniels and Hughes.

**Results:**

A total of 346 HbA1c-mortality-pairs from 205 single randomized trials were included in the analysis. Regarding the risk difference of all-cause mortality, there was no evidence for surrogacy of the HbA1c value. For the log relative risk, a small positive association between HbA1c and the all-cause mortality estimate (slope 0.129 [95% confidence interval −0.043; 0.302]) was observed. However, there was no sign of valid surrogacy.

**Conclusions:**

Based on the results of more than 200 randomized trials, HbA1c is not a valid surrogate marker for all-cause mortality in people with type 2 diabetes.

## Introduction

Between 2003 and 2012, all 26 trials for diabetes treatment that led to approval by the FDA used the surrogate marker HbA1c as the primary efficacy endpoint [1]. In seven of these trials, the rationale for using HbA1c as a surrogate was discussed, and three of them justified using it because the respective evidence “corresponds to the highest level of evidence for surrogacy using the ICH-9 (International Council for Harmonization of Technical Requirements for Pharmaceuticals for Human Use) criteria” [1].

However, HbA1c has also been repeatedly questioned as a valid surrogate marker. While it is widely accepted that hyperglycemia as measured by HbA1c is a risk factor for macro-and microvascular diabetes complications, this does not necessarily mean that actively lowering HbA1c also comes with benefits in clinical and patient-relevant outcomes. For example, there were a number of glucose-lowering drugs that had been retracted from the market (troglitazone [2], muraglitazar [3], or rosiglitazone [4]) due to severe clinical adverse events, although their glucose-lowering effect was without doubt and proven in well-conducted randomized trials [5, 6]. Interestingly, we also found the opposite case [5] where a glucose-lowering drug (metformin) with a rather modest effect in terms of HbA1c decrease yields an unexpectedly large cardiovascular benefit and is currently even considered an anti-aging treatment [7].

In addition, randomized trials that assessed intensified glycemic control strategies targeting fixed HbA1c levels (ACCORD [8], ADVANCE [9], VADT [10]) did not show convincing results for all clinical outcomes. Instead, there were signals for increased rates of severe hypoglycemia or all-cause mortality [5, 6].

Systematic empirical evidence on the surrogate validity of HbA1c for patient-relevant outcomes is scarce. Indeed, we are only aware of two meta-analyses that assessed the surrogacy for all-cause and cardiovascular mortality or clinical outcomes as primary endpoints [11, 12].

From a methodological point of view, the ICH Harmonized Tripartite Guideline on “Statistical Principles for Clinical Trials” [13] requests for a surrogate endpoint to be valid that (1) the surrogate is a prognostic marker for the clinical outcome in epidemiological studies and (2) treatment effects on the surrogate reliably predict treatment effects on the clinical outcome. With respect to (2) Daniels and Hughes [14] further operationalized this requirement and proposed to investigate whether when regressing the surrogate on the clinical outcome, (2a) the slope of the regression line should be different from zero, (2b) the intercept from the regression should be zero (otherwise, a treatment difference on the clinical scale would correspond with the absence of a treatment effect on the surrogate scale), and (2c) the correlation between surrogate effects and clinical outcome effects should be 1.

There is solid evidence for the prognostic value of HbA1c for all-cause mortality from epidemiological studies, which had already been collected in a large systematic review [15]. But the results of randomized trials have hardly been summarized with this question in mind. Rivera et al. recently reported the results of a systematic review of randomized trials with trial-level meta-analysis. They found no evidence to support the use of HbA1c as a surrogate for all-cause mortality in people with type 2 diabetes [12]. However, the restrictive, somewhat arbitrary inclusion criteria (at least 1000 participants and 20 events of clinical outcomes per trial, follow-up and/or duration of intervention of at least 52 weeks) in the meta-analysis on all-cause mortality led to the inclusion of only 19 cardiovascular outcome trials (CVOT) compromising the representativeness of the results. Moreover, solely the correlation between HbA1c and all-cause mortality, i.e., our criterion 2c, was used to assess surrogacy. Therefore, we present here an in-depth investigation of the validity of HbA1c in terms of the criteria of Daniels and Hughes [14] and systematically compare the treatment effects on HbA1c and the all-cause mortality scale as available from a large number of randomized trials.

## Methods

In terms of available trials, we relied on the systematic review of Palmer et al. [16] which included parallel-group randomized clinical trials that compared two individual glucose-lowering drug classes or placebo for the treatment of type 2 diabetes and had been published up to March 2016. The available drug classes were metformin, sulfonylurea, thiazolidinedione, DPP-4 inhibitor, SGLT2 inhibitor, GLP-1 receptor agonist, basal insulin, meglitinide, and α-glucosidase inhibitor. In addition, treatment had to be given for at least 24 weeks.

We obtained the full texts of all trials from the Palmer review, and extracted the available information on all-cause mortality, HbA1c at baseline and after treatment, and further description on drugs, sample sizes, and baseline characteristics of the trial populations. Extraction was performed by a single reviewer (W.S.), and double-checked by a second (T.F. or A.L.), remaining discrepancies were discussed with a fourth reviewer (O.K.) until consensus was reached. In addition, we validated our results by referring to the respective figures in the online supplement of Palmer et al. Numerical information from graphical displays was extracted by using WebPlotDigitizer, version 4.2 [17].

To achieve the most recent information from all trials, we also contacted the corresponding authors per email (plus a single reminder email after three to seven weeks) and asked for follow-up studies or additional information on all-cause mortality.

Furthermore, we performed citation tracking and downloaded all citations of all randomized trials from the Web of Science (http://www.isiknowledge.com) between August and November 2017. These citations (plus their abstracts) were searched for the name of the first author of the respective trial or the term “follow-up” to find additional follow-up information.

As the study does not include personalized, but only publication-based data, we did not seek for a vote of an ethics committee.

For studies with more than two treatment groups, we defined a reference group (if present the placebo group, in other cases the group with a lower dosage, the group with the more established, older treatment, or the group with the lower number of treatments for combination therapies), and computed effect estimates for each group in comparison to this reference group. As such, more than one pair of HbA1c and all-cause mortality treatment effects could originate from a single trial.

Information on HbA1c was available in various forms at baseline and after treatment (mean or change from baseline, standard deviations, standard errors, confidence intervals (CI), p-values, etc.), and we used the recommendations of the Cochrane collaboration [18] to arrive at a treatment effect estimator for HbA1c and its corresponding standard error. In any case, we always used the baseline information to adjust the HbA1c effect for baseline differences and give the final treatment effect for HbA1c as a difference. For this task, we also had to use a single multiple imputation step to impute 8% of missing values for the HbA1c standard error. If information on follow-up studies was available, we always used this one and discarded the results from the original publication.

To measure the treatment effect in terms of all-cause mortality, we computed risk differences (RD) as well as log relative risks (LogRR). As there were numerous trials without any observed deaths, for which case the relative risk is undefined in its standard form, we estimated the LogRR by using the median-unbiased estimation principle as proposed by Carter et al. [19]

For analysis, we only included trials that had information on treatment effects for HbA1c as well as for all-cause mortality. HbA1c differences as well as RD and LogRR for all-cause mortality were scaled so that a negative value indicates a benefit for the experimental treatment.

To measure actual surrogacy, we computed weighted linear regression models with a random intercept for the study effect. The all-cause mortality estimate was defined as the outcome in these models and the estimate for HbA1c difference as a fixed effect covariate. To account for the fact that estimates were assessed with different precisions, we referred to the standard idea of inverse-variance weighting and used the inverse of the estimation variance of the HbA1c difference as a weighting factor in the analysis. We ignored the differing estimation uncertainty in the all-cause mortality estimates because those estimates and their estimation variances originate from very low numbers of events, and we foresaw extreme weights potentially compromising the analysis. By using a random intercept term for the study effect, we adjusted for the fact that some pairs of effect estimates originate from the same trials and are therefore expected to be correlated. Then we assessed whether surrogacy was moderated by the baseline properties of the trials’ samples. We fitted separate models with an additional interaction term of the HbA1c difference and trial size, age at baseline, percentage of males, duration of diabetes, HbA1c at baseline, BMI at baseline, or trial duration. Finally, we determined the correlation between HbA1c difference and all-cause mortality by Pearson's correlation coefficient and the related 95% CI (Fisher). All analyses were conducted with SAS, version 9.4 (SAS Institute, Cary, NC, USA).

## Results

We achieved the full texts of 297 trials. Two trials from the Palmer sample were not eligible due to language reasons, three due to logistic reasons, and one trial had been retracted. In terms of gaining additional mortality information, we contacted the corresponding author of 243 trials, and achieved 63 (corresponding to 26%) responses. Citation tracking via “Web of Science” could be performed for 288 RCTs.

For the final analysis, we were able to use 346 pairs (i.e., with information on the treatment effect for HbA1c as well as for all-cause mortality) of effect estimates from 205 single trials. Follow-up information could be included for 20 pairs from 13 trials. The 346 pairs of effect estimates were informed by 361 deaths from 122,245 observations, the mean (median) trial duration was 41 (26) weeks, and 120 pairs originated from observation periods of one year or longer.

The mean/median HbA1c difference across the 346 estimates was − 0.488/− 0.500%-points) (corresponding to − 5/− 5 mmol/mol), and 277 HbA1c differences were smaller than zero, indicating that experimental treatments in general led to larger benefits in glucose lowering. The mean/median RD was 0.001/0%-points, and the mean/median LogRR was − 0.036/− 0.006, indicating the absence of benefit for the experimental treatment in terms of all-cause mortality.

**Fig. 1:**
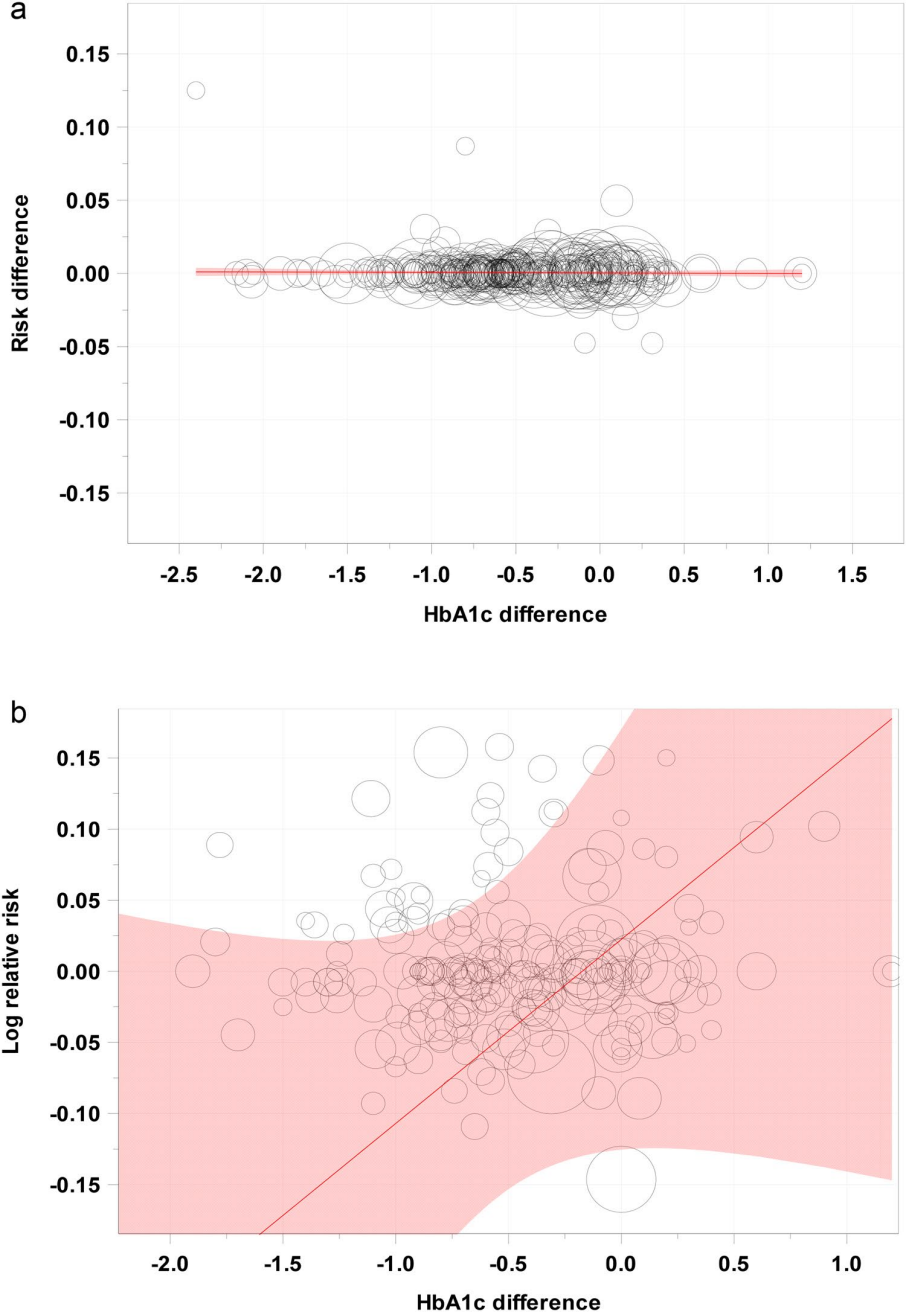
Scatterplot of the pairs of treatment effect estimates for HbA1c (x-axis) and all-cause mortality (y-axis) with the estimated regression line from the weighted linear regression model. **a** Risk difference, **b** Log relative risk. The size of the bubbles corresponds to the weight in the weighted linear model. The shaded areas give the pointwise 95% confidence intervals

In Table 1 and Fig. 1, we provide the results from the weighted linear regression models. In terms of the RD, there was no association between HbA1c and the mortality effect and the narrow 95% CIs definitely excluded all clinically useful values for surrogacy. Regarding the LogRR, there was a small positive association, but the large 95% CI potentially also allows for negative association, so there is also no clear sign of valid surrogacy. The interaction analyses indicated no evidence of effect moderation (Table 2). To conclude, none of the three surrogate criteria of Daniels and Hughes was met, both in terms of the RD and the LogRR.

**Table 1:** Results from the weighted linear regression and weighted correlation analysis with respect to the surrogacy criteria as given by Daniels and Hughes [14]

Parameter	Estimate	95% Confidence interval
*Risk difference*		
Slope (Crit. 2a)	− 0.031%	[− 0.179%; 0.117%]
Intercept (Crit. 2b)	0.036%	[− 0.076%; 0.148%]
Correlation (Crit. 2c)	− 0.089	[− 0.232; 0.060]
*Log relative risk*		
Slope (Crit. 2a)	0.129	[− 0.043; 0.302]
Intercept (Crit. 2b)	0.022	[− 0.125; 0.170]
Correlation (Crit. 2c)	− 0.010	[− 0.145; 0.134]

The parameter estimates and the respective 95% confidence intervals are given

**Table 2:** Results from the subgroup analyses to assess whether surrogacy was moderated by baseline properties of the trials’ sample models

Parameter (Interaction of HbA1c difference and …)	Estimate	95% Confidence interval
*Risk difference*		
Sample size	0.00054%	[− 0.00017%; 0.00124%]
Age at baseline	− 0.01501%	[− 0.05964%; 0.02962%]
Percentage of males	− 0.00422%	[− 0.01977%; 0.01133%]
Duration of diabetes	− 0.00317%	[− 0.03803%; 0.03169%]
HbA1c at baseline	− 0.07969%	[− 0.39406%; 0.23469%]
BMI at baseline	0.00407%	[− 0.03300%; 0.04114%]
Trial duration	− 0.00274%	[− 0.01123%; 0.00575%]
*Log relative risk*		
Sample size	0.0004	[− 0.0004; 0.0013]
Age at baseline	− 0.0038	[− 0.0514; 0.0438]
Percentage of males	− 0.0115	[− 0.0236; 0.0006]
Duration of diabetes	0.0125	[− 0.0458; 0.0707]
HbA1c at baseline	− 0.1341	[− 0.3676; 0.0994]
BMI at baseline	− 0.0176	[− 0.0719; 0.0367]
Trial duration	− 0.0058	[− 0.0145; 0.0029]

The parameter estimates (with 95% confidence interval) of the interaction term of the HbA1c difference with the respective potential moderators are given

## Discussion

With reference to the surrogacy criteria of Daniels and Hughes [14], HbA1c is no valid surrogate marker for all-cause mortality in diabetes research. This result was found in a meta-analysis including 346 pairs of effect estimates from 205 different randomized trials that assessed a multitude of pharmacological treatments for type 2 diabetes and was informed by more than 120,000 observations.

### Comparison with previous findings

The LogRR estimated in our study (0.129 [95% CI − 0.043; 0.302]) is in good accordance with previous work from a smaller sample where the log odds ratio (LogOR) for all-cause-mortality was 0.222 (standard error: 0.168, corresponding 95% CI [− 0.107; 0.551]) [11]. In addition, LogRRs are closer to 0 than LogORs enhancing the similarity of both results. Consistent with the recently published meta-analysis by Rivera et al., we found no convincing evidence for surrogacy of HbA1c in terms of mortality [12]. The differences in LogRRs and correlation coefficients between the study by Rivera et al. (LogRR 0.320 [95% CI − 0.151; 0.791]; Pearson's correlation coefficient 0.328 [− 0.148; 0.681]) and our present analysis (LogRR 0.129 [− 0.043; 0.302]; correlation − 0.010 [− 0.145; 0.134]) might be attributable to methodological differences, differences in the inclusion criteria or publication periods of the trials. Our results furthermore correspond with common observations from clinical and regulatory reality [5, 6].

In contrast, in a current systematic review with meta-analysis a decrease in the incidence of major adverse cardiac events (MACE) including cardiovascular deaths on various antidiabetic drugs was found [20]. This decrease correlated with HbA1c reduction (*r* = 0.88 [95% CI 0.67; 0.96]) and persisted even after adjustment for weight loss. However, the study was based solely on CVOT. One of the main issues criticizing CVOT is the selection of the study population. Eligibility requirements for study participation usually include having a history of cardiovascular disease or high cardiovascular risk in addition to certain age and blood glucose control specifications. According to estimates from population-based data, only 4% to 38% of the real-world population with type 2 diabetes would have qualified for enrollment into renowned CVOTs [21–23]. Moreover, as pointed to by Lipska/Krumholz [24], CVOTs would probably not be very informative for the present study question, because the study protocols of the CVOTs in general encourage additional glucose control according to the local guidelines. As such, HbA1c effects from CVOTs cannot be attributed completely to the treatments under study, but will be confounded by other glucose-lowering therapies. Any convincing effects on mortality in the CVOTs are, thus, most likely unrelated to the glucose-lowering effect of the compounds under study.

### Explanatory approaches

The lack of surrogacy of HbA1c for mortality found in the current meta-analysis can be explained by several circumstances. First, the long-standing focus on glycemic control as an established primary endpoint in diabetes treatment can be attributed to the fact that HbA1c values were generally larger in former times and cardioprotective therapies, e.g., statins, were not used that widespread [24]. As such, parallels of HbA1c improvement with improvements in cardiovascular endpoints could be expected and were actually seen in the large trials (DCCT, UKPDS) that assessed intensive glycemic control. However, it is possible that these beneficial effects are not due to glucose lowering but rather to other concomitant treatments, such as antihypertensive agents [11, 25]. Second, as known from the ACCORD and ADVANCE trials, achieving good blood glucose control could be at the cost of the number of hypoglycemic events and weight gain and thus not result in reduced mortality [25, 26].

### Strengths and limitations

The results of this study are based on the findings of over 200 international randomized trials enrolling more than 120,000 participants. It, thus, provides a much more comprehensive overview of the current evidence, when compared to previous studies that included always less than 20 trials. Especially, smaller studies and studies of shorter duration were also taken into account. The application of an advanced statistical method (median-unbiased estimation principle) made it possible to include not only studies with a small number of outcome events but even those without any events.

We restricted here to the outcome of all-cause mortality, since we believe that this outcome is of highest relevance for the patients. Furthermore, all-cause mortality can be measured with high validity and reliability and is also not influenced by competing risk as other major, non-fatal cardiovascular events. In addition, we made considerable efforts to update the mortality information from the single trials as we were foreseeing low number of deaths in the trials. To be concrete, we performed a systematic citation tracking of the initial trial publications and contacted their authors. Finally, consistent with the recommendations on participatory decision-making [27], we used both an absolute and a relative effect measure.

It is certainly a limitation of our analysis that the average observation time in our study sample was rather short, resulting in a low absolute number of deaths across all trials. It might be possible that treatment effects on all-cause mortality may be seen with longer observation times. Having actually anticipated this, we contacted the authors of our randomized trials to achieve additional information on long-term follow-up. However, this was less successful than expected, and little follow-up information was gained overall.

Another limitation is that we did not include the results of the large cardiovascular outcome trials (CVOTs), which force pharmaceutical industry today to use cardiovascular instead of glucose-lowering endpoints if seeking regulatory approval. The limitations of these trials with regard to the current research question have already been described above.

We acknowledge as a final limitation of our study (see also [11]) that we only used aggregate data on the trial level to assess surrogacy of HbA1c. This comes with the danger of ecological bias. More precisely, it might be possible that actual surrogacy could be observed when individual patient data would have been available. It might, thus, have been worthwhile (or an avenue for further research on that issue) to seek to receive individual patient data from the trials. However, it is not guaranteed that all authors would like to share their data. For example, only roughly a quarter of all authors answered to our simple question on follow-up information, and sharing original data would mean a tremendously larger amount of work and commitment.

Future studies may also analyze observational studies focusing on associations between HbA1c and all-cause mortality to complement knowledge and obtain information independent of treatment effects.

## Conclusion

Based on the evidence of numerous randomized trials, we conclude that HbA1c is not a valid surrogate marker for all-cause mortality in people with type 2 diabetes. Our results strengthen the rationale that clinical decisions should not only be guided by glycemic parameters, but should also take into account patient-relevant outcomes [12, 24].
